# ANALYSIS OF DEATH AND PALLIATIVE CARE IN A NEONATAL INTENSIVE CARE UNIT

**DOI:** 10.1590/1984-0462/;2017;35;2;00012

**Published:** 2017-05-15

**Authors:** Ligia Marçola, Silvia Maria Macedo de Barbosa, Ivete Zoboli, Rita Tiziana Verardo Polastrini, Maria Esther Jurfest Ceccon

**Affiliations:** a Instituto da Criança do Hospital das Clínicas da Faculdade de Medicina da Universidade de São Paulo (USP), São Paulo, SP, Brasil.

**Keywords:** newborn, palliative care, Neonatal Intensive Care Units

## Abstract

**Objective:**

To characterize cases of children admitted to the Neonatal Intensive Care Unit of a tertiary university hospital who died in the period ranging from January 01, 2012 to July 31, 2014, and who required palliative care and/or were subjected to it.

**Methods:**

A retrospective descriptive study was carried out by reviewing the medical records of these patients to collect data and to perform descriptive statistical analysis.

**Results:**

During the study period, 49 children died after at least 48 hours from the time of admission. Of those, 18% children were extremely premature infants and 77% children had malformations. Although necessary for all of the patients in this study, palliative care was provided for only 20% of patients who died. Among the 12 babies who were not resuscitated, 33% of babies were not in palliative care. The Pain and Palliative Care Unit of the institution followed only four neonates in palliative care. These patients were using many invasive devices, had high therapeutic investment, and also altered pain scale scores.

**Conclusions:**

This study exhibited a large proportion of newborn infants with serious diseases and health conditions. In a few cases the patients received palliative care, but most of them were not even discussed under palliative point of view. We hope that this study will call attention to the need to propose protocols and implement training for the best treatment of these children.

## RESUMO

**Objetivo:**

Caracterizar as crianças admitidas na Unidade de Terapia Intensiva Neonatal de um hospital universitário terciário que foram a óbito no período de 1.º/01/2012 a 31/07/2014 e que necessitavam de cuidados paliativos e/ou para as quais foram indicados tal tipo de cuidado.

**Métodos:**

Realizou-se estudo descritivo retrospectivo. Foi feita revisão dos prontuários desses pacientes para coleta de dados e análise estatística descritiva.

**Resultados:**

No período estudado 49 crianças morreram após, no mínimo, 48 horas de internação. Destas, 18% eram prematuros extremos e 77% apresentavam malformações maiores. Apesar de necessários a todos, cuidados paliativos foram realizados apenas em 20% dos casos de óbito. Dos 12 bebês que não foram reanimados, 33% não estavam em cuidados paliativos. A Unidade de Dor e Cuidados Paliativos da instituição acompanhou somente quatro crianças que ficaram em cuidados paliativos. Foram observados uso de muitos dispositivos invasivos e alto investimento terapêutico, além de escores de escala de dor alterados.

**Conclusões:**

Detectou-se neste estudo grande proporção de recém-nascidos com doenças e condições graves de saúde. Em alguns poucos casos foram estabelecidos cuidados paliativos, porém para a maior parte não foi nem discutido tal cuidado. Espera-se que este trabalho chame a atenção para a necessidade da proposição de protocolos nessa unidade e capacitação de equipes para o melhor tratamento dessas crianças.

## INTRODUCTION

Owing to advances in science and technology over the last 50 years, many children with rare diseases and complex clinical conditions such as congenital malformations and prematurity are achieving higher survival rates.^1^ ^,^ ^2^ ^,^ ^3^ Nevertheless, morbidities with consequences for future life are progressively increasing, which brings to the fore the need to examine the therapeutics and treatments implemented.^1^ ^,^ ^4^ ^,^ ^5^

Western culture has difficulties coping with death, especially the death of children. For the family, the loss of a child has greater impact than the loss of an adult member.^2^ As for the medical team, it is common for a fatal prognosis to lead to a sense of failure.^2^ ^,^ ^5^ ^,^ ^6^ When the death of a child approaches, conflicts arise and there is a need to discuss legal and ethical issues.^5^ ^,^ ^7^ ^,^ ^8^

Palliative care is very important in this delicate context, as it seeks to improve the quality of life of patients who face diseases that threaten or limit their lives and the quality of life of their families. Such care does not extend only to patients with terminal illnesses; patients with severe illness at any stage can benefit from this type of care as well.^1^ ^,^ ^2^ ^,^ ^7^ With its comprehensive approach, palliative care can make a potentially destructive experience become one that strengthens family bonds and allows the professionals involved to treat and comfort whenever possible, even if there is no cure for the disease.^2^ ^,^ ^7^

According to the Department of Informatics of the Unified Health System (DATASUS), 26,730 neonatal deaths occurred in Brazil in 2013.^9^ The main causes include complications related to prematurity and low birth weight, congenital malformations, sepsis, and other complications at the time of childbirth.^1^ ^,^ ^9^ In parallel, the main situations described in the literature on palliative care involve cases of extremely premature infants with complex, severe asphyxiated malformations that no longer present conditions for cure and/or poor quality of life in the near future.^7^ ^,^ ^8^ Therefore, as most of the Brazilian newborns that die have conditions for which palliative care would be beneficial, it becomes relevant to discuss and investigate this issue at a national level.

Despite the importance given to the subject by international agencies and growing worldwide concern, the literature exhibits few descriptions of palliative care protocols and programs used in Neonatal Intensive Care Units (NICUs).^6^ ^,^ ^10^ In Brazil, there is a lack of information and studies for this age group, which demonstrates a need for more research. Considering this larger context, the objectives of this study were to detect and characterize children admitted to a Neonatal Intensive Care Center (NICC) who required palliative care and/or were undergoing palliative care when they died.

## METHOD

The research was carried out at the Children’s Institute of the Neonatal Intensive Care Center 2 (NICC-2), part of the Hospital of the Clinical Complex of the Medical School of the Universidade de São Paulo, located in São Paulo. This is a retrospective descriptive study in which the data were obtained by means of a review of medical records. We analyzed the following: pre- and postnatal diagnoses and causes of death, cardiopulmonary resuscitation, presence of palliative care and return to curative treatment, documentation of multidisciplinary meetings, and meetings with parents. On the day of death, the following data were collected: use of medications, therapeutic investment, and use of invasive devices. There were nine devices: bladder catheter; orogastric or nasogastric tubes; central venous catheters; catheters for peritoneal dialysis; peripheral venous access; oral or nasal cannula; tracheostomy; gastrostomy; and ileostomy and colostomy. In addition, we analyzed the follow-ups carried out by other professionals, as well as the application of the Neonatal Infant Pain Scale (NIPS)^11^ and the values ​​found, with values greater than or equal to three serving as an indication of the presence of pain.

The inclusion criterion in the study was newborns admitted to NICC-2 who died between January 1, 2012 and July 31, 2014. The exclusion criterion consisted of death within the first 48 hours of hospitalization.

The analysis was carried out using descriptive statistics. The research began after approval of the Research and Ethics Committee of the department involved, the institution’s Ethics and Research Commission (CAAE 48186515.4.0000.0068; Opinion no. 1215196, September 4, 2015).

## RESULTS

During the study period, 60 deaths occurred among children who were hospitalized at NICC-2. Of these, 18% (11 children) died within the first 48 hours of hospitalization and were not included in the study. In other words, 49 (82%) children died after at least 48 hours from the admission. The age at death ranged from 3 to 376 days (mean of 49 days). In 84% of cases, admission to NICC-2 occurred in the neonatal period, with 41% of all 49 newborns admitted on the first day of life. The mean length of hospital stay was 39 days.

We found 38 children with major malformations (77% of all deaths): congenital diaphragmatic hernia, complex cardiac malformations, holoprosencephaly, esophageal atresia, duodenal atresia, gastroschisis, omphalocele, bilateral renal hypoplasia, prune belly syndrome, cystic adenomatoid malformation, osteogenesis imperfecta, and malformations of xiphopagus conjoined twins. Of these 38 patients, 12 (31%) had genetic syndromes. Characterization of the sample is found in Table 1.

**Table 1:**
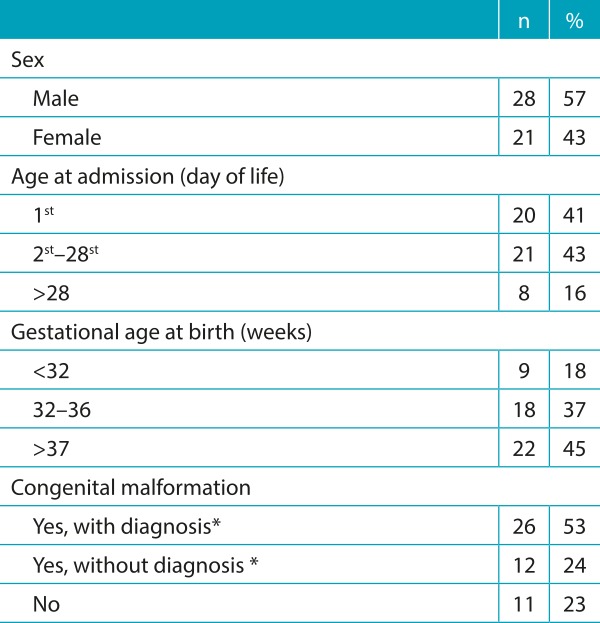
Characterization of the 49 patients.

*Prenatal diagnosis.

With respect to the causes of death, neonatal sepsis was the main cause in 61% of deaths, followed by cardiogenic shock in 17%, respiratory failure in 8%, hemorrhagic shock in 4%, and pulmonary hemorrhage in 4% of deaths. The other 6% of deaths occurred due to acute renal failure, cardiac tamponade, and brain death.

Of the patients who died, 75% underwent resuscitation maneuvers; of the 25% who were not reanimated, 33% did not receive palliative care. Two children referred in palliative care were submitted to resuscitation on the day of death.

Of the 49 patients who died, only 10 (20.0% of all cases) were in palliative care (Table 2). In 3 of the 10 cases, curative efforts were resumed (two by decision of the attendant and one at the request of the family). In only five patients, the care strategy was discussed at a multidisciplinary meeting. Four patients received followed up care from the pain and palliative care group and six from psychology.

**Table 2:**
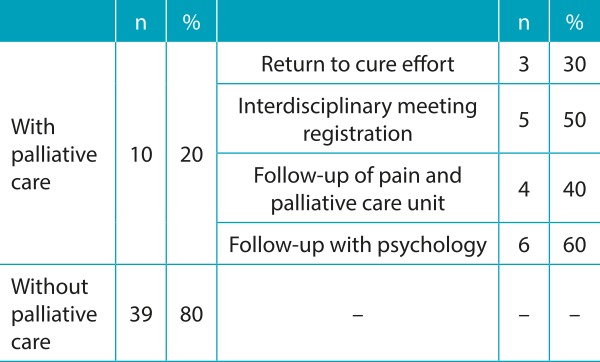
Indication and performance of palliative care.

Of all the cases of death, only eight were discussed in a multidisciplinary meeting from the palliative point of view; all patients received indication of palliative care, which means that two patients receiving this care did not have this type of discussion recorded in the medical record.

The use of many invasive devices and high therapeutic investment was observed. The pain scale was applied in all children, several with altered scores, most of them being medicated with sedatives and/or analgesics (Tables 3 and 4).

**Table 3:**
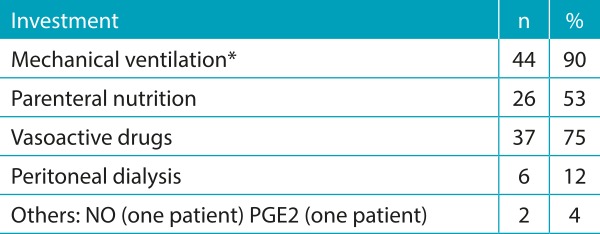
Therapeutic investment on the day of death in the 49 newborns evaluated.

*Two patients received high-frequency ventilation; NO: nitric oxide; PGE2: prostaglandin E2.

**Table 4:**
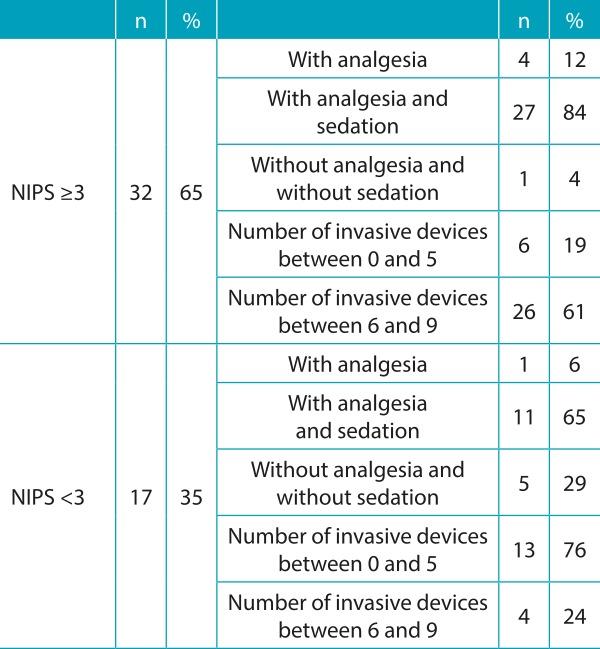
Pain, analgesia, and invasive devices in the 49 newborns assessed on the day of death.

NIPS: Neonatal Infant Pain Scale.

## DISCUSSION

Neonatal palliative care is increasingly being devised, considered, and implemented. There is still a long way to go in the midst of ethical, human, and operational dilemmas and challenges, but there is increasing concern worldwide^4^ ^,^ ^5^ ^,^ ^8^ and an ever-growing demand for its use.

The situation is no different in the NICC analyzed in the study - a baby nursery that receives newborns and children aged up to 45 days -, with clinical cases and an expressive number of surgical patients, which serves as a national reference. In this health center, many infants were in need of palliative care and in some cases did indeed receive it, though its implementation did not always involve the intervention of the institution’s Pain and Palliative Care Unit. Most of the patients who died were admitted to this NICC in the first days of life and had prolonged hospitalization; however, many of them already had prenatal diagnosis of malformations. Therefore, it would have been possible to establish an early connection and initiate palliative care more comprehensively. These discoveries demonstrate a need to broaden and improve the implementation of palliative care.

We identified nine patients (18% of the study group) with a gestational age of less than 32 weeks, a condition that already presents a high risk of death and morbidities, and therefore justifies the need for palliative care for this population.^10^ ^,^ ^12^ In countries such as the Netherlands, extreme prematurity is the second cause of indication for neonatal palliative care.^10^ That being said, of the nine premature infants in this study only one (11%) received this care.

We observed 77% prevalence of major malformations in the evaluated group - probably attributed to the fact that the health center is a reference service for such pathologies -, representing a situation in which patients would benefit from palliative care, regardless of the prognosis itself. Among all these patients with malformations, 68% had prenatal diagnosis. In the literature, up to 90% of prenatal diagnosis of congenital anomalies is found.^12^ ^,^ ^13^ This demonstrates the need for better quality prenatal care in health services. It is noteworthy that, in addition to allowing better planning of pregnancy and delivery, this early diagnosis allows for the initiation of perinatal palliative care.^1^ ^,^ ^10^ ^,^ ^12^ ^,^ ^14^

According to DATASUS data, the causes of death in the study population are similar to those found throughout Brazil. Cases were divided into groups according to the International Classification of Diseases and Related Health Problems 10 (ICD-10).^9^ In this study, Group P refers to causes associated with the neonatal period, including neonatal sepsis, and accounts for 74% of cases. Group R of ICD-10 refers to circulatory and hypovolemic shock and accounts for 20% of cases in this study. All cases presented malformations and were therefore grouped in the Q Group of ICD-10, the second cause of neonatal death in Brazil. The remaining 6% of deaths were due to less frequent causes in Brazil (acute renal failure, cardiac tamponade, and brain death).

There is a contradiction in the fact that some children who were not reanimated were not in palliative care. It can be understood that they were not reanimated because they were considered terminal; however, this would represent a precise indication of such care, as not every patient in palliative care is terminal, but every terminal patient should receive palliative care.^1^ ^,^ ^2^ ^,^ ^8^ It is also worth reiterating that only eight (16%) of the children who died had a record of multidisciplinary meetings for the discussion of their case from a palliative point of view - an important event to consider this type of care and to limit or even prevent potential conflicts.^3^ ^,^ ^10^ In countries where discussion and palliative care are more advanced, up to 87% of children who die in NICUs have the case discussed in relation to end-of-life decisions and therapeutic investment.^3^ ^,^ ^12^ However, there remains significant need for further development and advancement of this care, especially in Latin American countries.^12^ ^,^ ^14^

As is common in most retrospective studies, one limitation of this study was the availability of information. The collection and analysis of the data depended on the recording of the information, which was not always complete and/or legible. Thus, there may have been some bias in the analysis of the data obtained.

In conclusion, the quality of death of the children in this study proved to be very poor. The day of death exhibited high therapeutic investment, overuse of many invasive devices and poorly controlled pain. There is a need to expand and improve the application of palliative care in this service, since not all of those who need this strategy had access to it, and among those who are able to access palliative care, it is often not carried out properly due to lack of multidisciplinary discussion and limited involvement of the Pain and Palliative Care Unit.

There are few descriptions in the literature of palliative care protocols used in NICUs. The greatest difficulty documented relates to the indication of such care. When diagnosing the situation in this NICU, this study draws attention to the need for palliative care protocols and programs, as well as the training of the care team so that the children treated have the best and most dignified treatment possible, even if a cure is not achievable.
